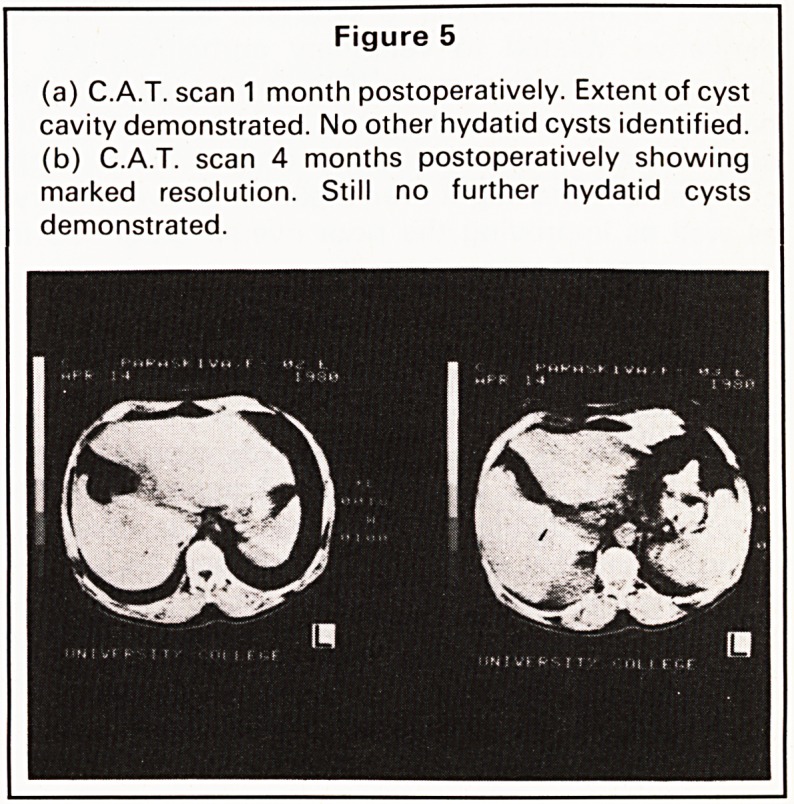# Liver Hydatid Cyst Rupture

**Published:** 1983-07

**Authors:** N. I. Ramus, T. Lyons

**Affiliations:** Department of Surgery, Bristol Royal Infirmary; Department of Surgery, Bristol Royal Infirmary

**Keywords:** Hydatid cyst, Liver, Obstructive jaundice, rupture

## Abstract

We report two cases that demonstrate not only rare presentations of a rare disease, but accepted methods of hydatid management and the use of C.A.T. scanning in diagnosis and follow-up. They also confirm the value of operative cholangiography and perhaps introduce the use of a new anthelminthic agent, mebendazole, in association with surgery.


### Liver Hydatid Cyst Rupture

**N. I. Ramus, M.D., F.R.C.S. and T. Lyons, M.B., Ch.B.**
Department of Surgery, Bristol Royal Infirmary

Keywords: Hydatid cyst; Liver; Obstructive jaundice, rupture

### SUMMARY

We report two cases that demonstrate not only rare presentations of a rare disease, but accepted methods of hydatid management and the use of C.A.T. scanning in diagnosis and follow-up. They also confirm the value of operative cholangiography and perhaps introduce the use of a new anthelminthic agent, mebendazole, in association with surgery.

### INTRODUCTION

Hydatid disease is still uncommon in Great Britain. One hundred and seventy cases were reported to laboratories in England and Wales between 1966, when recording first began, and 1980.[1] The liver is the site of predilection in more than 60% of reported cases.[1,2] It is probably involved at some stage in every case as entry into the portal vein is part of the life cycle of *Echinococcus granulosus.* Obstructive jaundice from intrabiliary rupture of a hydatid cyst of the liver is therefore very rare in this country, but described in 16% of patients in Kattan's series from Iraq.[2] Intraperitoneal and intrathoracic rupture of the cyst were even rarer, occurring in only 4.2% of cases.[2]

We present two cases of hepatic hydatid cysts, one presenting with obstructive jaundice and the other as intraperitoneal rupture following a road traffic accident.

### Case report 1

A 28-year-old male Spaniard presented with intermittent obstructive jaundice, pain and fever. Examination revealed hepatomegaly, with a discrete mass in the right lobe. Hydatid complement fixation test (H.C.F.T.) was positive, and plain abdominal radiographs showed calcification within both lobes of the liver. Computerised axial tomography (C.A.T. scan: Figure 1) confirmed a large fluid-filled cyst with multiple septa in the right lobe and a smaller calcified cyst in the left. During investigation, the mass became smaller, but an intravenous cholangiogram (I.V.C.) demonstrated only medial displacement of the biliary tree, without any evidence of obstruction or filling defects.

**Figure 1**

C.A.T. scan demonstrating fluid-filled cyst with multiple septa in right lobe of the liver and a smaller calcified cyst in the left.

At operation a tense cyst of the right lobe was confirmed (Figure 2) with a small calcified cyst in the

*Address for correspondence*: Mr. N. I. Ramus, Bristol Royal Infirmary, Bristol BS2 8HW.

**Figure 2**

Laparotomy findings with gallbladder displaced by large liver cyst with thinning of liver substance over its surface.

left lobe. Using 1% cetrimide as a scolicide[3] the abdominal cavity was packed off, and the cyst was decompressed with minimal spillage by means of a prearranged funnel-shaped sucker.[4] Dissection of the ectocyst from the liver demonstrated a bile duct communication from which a daughter cyst was seen to escape. Operative cholangiography through both this communication and a needle in the common bile duct (Figure 3) confirmed a side-hole in the right hepatic duct which was accordingly sutured. As no further hydatid material was identified in the biliary tree and there was free flow into the duodenum, the common bile duct was not explored. Similarly, the gallbladder was not removed. The cyst cavity was irrigated with silver nitrate solution[3,4] and the omentum was inserted into the defect. A corrugated drain was left in situ. Cetrimide lavage followed removal of the packs. Postoperative recovery was uneventful with the drain removed at 10 days. Six months later he was well and returned to Spain.

**Case report 2**

A previously healthy 36-year-old Greek male, resident in England for 19 years, was seen following a road traffic accident in which he received blunt trauma to the upper abdomen. On examination he was pale, sweating and restless. Blood pressure was 110/70 mm/Hg and pulse 70, regular but weak. There was marked abdominal tenderness with guarding and superficial bruising over the right hypochrondrium. Bowel sounds were absent. Haemoglobin and white cell count (WCC) were normal. Chest and abdominal radiographs were unremarkable.

At emergency laparotomy for suspected intra-abdominal bleeding a large, ruptured, hydatid cyst of the right lobe of the liver was found. Approximately 1000 cc of blood together with many recognisable daughter cysts were lying free in the peritoneal cavity (Figure 4). Repeated peritoneal lavage was performed with 1% cetrimide as a scolicidal agent. The bleeding edges of the ectocyst were oversewn and the cavity was packed with omentum around a large tube drain.

Postoperatively he was treated with a new oral scolicidal agent, mebendazole, 800 mg three times a day, increased sequentially to 4.5 g daily, for 3 months. Initially, bile drainage from the cyst bed was 450 ml daily, but this ceased after 12 days.

His haemoglobin was 10.6 g/dl, WCC $17.1 \times 10^9/1$ with a 26% eosinophilia. Liver function tests revealed a marginally raised bilirubin (20 µmol/l), alkaline phosphatase (27 KA units) and aspartate transaminase (30 iu/l) which returned to normal after 45 days. Immediately postoperatively the serum H.C.F.T. showed a titre of 1 : 64; and the hydatid latex agglutination test (H.L.A.T.) was positive.

At discharge 4 weeks after admission the haemoglobin was 14.1 g/dl, WCC $7.8 \times 10^9/l$ with 2% eosinophils and serum H.C.F.T. titre of 1 : 2000. During follow-up he remained asymptomatic, the serum H.C.F.T. titre falling from 1 : 500 at 2 months to 1 : 16 at 9 months. Twenty months after rupture the H.C.F.T. was 1 : 32 but the H.L.A.T. remained positive. Serial C.A.T. scans have confirmed the continued presence of a marsupialised cyst but no new hydatid cysts have been demonstrated (Figure 5).

### DISCUSSION

With the advent of inexpensive international travel, previously uncommon diseases such as hydatid disease may be seen more frequently. Forty-three per cent of those cases reported between 1966 and 1980 were in patients from foreign countries.[1] Both our patients came from countries where hydatid disease is prevalent,[5] and one (Case 2) had also worked on a sheep farm in Cyprus.

The complications resulting from spontaneous and peroperative rupture of hydatid cysts of the liver include biliary obstruction, multiple peritoneal cyst implantation and anaphylactic shock.[6] Kattan, in 1977, found that although obstructive jaundice was often incomplete, the common bile duct was dilated and contained hydatid debris in all cases. Accordingly his surgical guide-lines included that the common bile duct must be explored and the ampulla dilated. In our case operative cholangiograms through a cyst communication to the right hepatic duct – the duct involved in 73% of cases[2] – and a

**Figure 3**

(a) Operative cholangiogram via a catheter passed into the cyst/bile duct communication confirming this to be a side hole in the right hepatic duct. The gallbladder has been dissected off the cyst and transposed medially. Multiple taped swab markers are noted. No hydatid debris has been demonstrated within the biliary tree. No duodenal flow is seen.

(b) Operative cholangiogram through a needle in the common bile duct after most of the taped swabs have been removed. The gallbladder has been returned to its anatomical position. Again, no hydatid debris demonstrated in the biliary tree, but free flow has occurred into the duodenum.

**Figure 4**

Hydatid material removed from the the peritoneal cavity. Daughter cysts easily recognisable.

**Figure 5**

(a) C.A.T. scan 1 month postoperatively. Extent of cyst cavity demonstrated. No other hydatid cysts identified.
(b) C.A.T. scan 4 months postoperatively showing marked resolution. Still no further hydatid cysts demonstrated.

needle in the common bile duct did not show hydatid debris. The common bile duct was therefore not explored.

Intraperitoneal rupture, particularly related to trauma, is very rare. Medvedev[7] reported the death of a previously fit young man from anaphylactic shock following the rupture of a hepatic hydatid cyst during a volleyball game. Marti et al.[8] described obstruction to the major and minor bile ducts by daughter cysts following intra-hepatic rupture of a hydatid cyst in a road traffic accident.

Our patient with intraperitoneal rupture was shocked but probably from hypovolaemia rather than anaphylaxis. Certainly no intraoperative problems were encountered. Postoperatively he drained bile for 12 days which was ascribed to an undiagnosed biliary cyst communication. He also had night sweats with an intermittent pyrexia thought to be related to his treatment with mebendazole[9] rather than a septic focus.

In both cases during the operations cetrimide was used as a scolicidal agent although for the elective procedure silver nitrate was also used. Both agents have been shown to be more effective than the traditional formalin.[3,4] Maximal precautions were taken to prevent spillage during the elective operation including a specially arranged funnel-shaped sucker to remove hydatid debris, an idea derived from the cryocone developed by Saidi.[4] In the second case, however, full peritoneal spillage had already taken place, hence the administration of the new systemic scolicidal agent, mebendazole. Until recently the only effective treatment for hydatid disease has been surgery[10] which is not without complication and may not be appropriate for disseminated disease, as in the present case. With the advent of mebendazole, a synthetic benzimidazole derivative, related to veterinary anthelminthics, a potentially effective medical treatment against the parasite has become available.[11] Although its efficacy is still under trial, this drug will certainly complement and might even replace elective surgery, as well as improving the poor overall prognosis in disseminated disease.

### ACKNOWLEDGEMENTS

We thank Mr. H. J. Espiner for permission to report Case 1 and the Hospital for Tropical Disease, London, in particular Dr. Bryceson for his advice as regards treatment with mebendazole and his follow-up of the patient in Case 2, and Mr. H. K. Bourns, F.R.C.S., previously consultant surgeon at Bristol Royal Infirmary, for his permission to report Case 2.

### REFERENCES

1. Public Health Laboratory Service. Communicable Disease Surveillance Centre (1972, 1977, 1981) Hydatid disease. Unpublished.
2. KATTAN, Y. B. (1977) Intra-biliary of hydatid cyst of the liver. *Ann.Roy.Coll.Surg.Engl.* **59**, 108–114.
3. MEYMERIAN, E., LUTTERMOSER, G. W., FRAYHA, G. J., SCHWABE, C. W. and PRESCOTT, B. (1963) Host–parasite relationships in echinococcosis. Laboratory evaluation of chemical scolicides as adjuncts to hydatid surgery. *Ann.Surg.* **158**, 211–215.
4. SAIDI, F. and NAZARIAN, I. (1971) Surgical treatment of hydatid cysts by freezing of cyst wall and instillation of 0.5 per cent silver nitrate solution. *The N.Engl.J.Med.* **284**, 1346–1350.
5. ROY, S. C., CHAKRAVARTTY, M., DAS, M. M. and CHATTERJEE, B. P. (1970) World incidence of hydatid disease in general and pulmonary hydatid disease in particular with special reference to India. *J. Indian M.A.* **55**, 212–217.
6. BAROSS, J. L. (1978) Hydatid disease of the liver. *Am.J.Surg.* **135**, 597–600.
7. MEDVEDEV, D. N. (1967) Death from anaphylactic shock due to the traumatic internal rupture of liver echinococcus. *Meditsinskaia Parazitologiia (Moskva)* **36**, 486–487.
8. MARTI, M. C. and RONNER, A. (1977) Traumatic rupture of hepatic hydatid cyst. *Helvetica Chirurgica Acta* **44**, 99–102.
9. Medical treatment for hydatid disease? Editorial (1979) *Br.Med.J.* **6190**, 563.
10. SAYEK, I., YALIN, R. and SANAC, Y. (1980) Surgical treatment of hydatid disease of the liver. *Archives of Surgery* **115**, 847–850.
11. MORRIS, D. L. (1981) Management of hydatid disease. *British Journal of Hospital Medicine* **25**, 586–595.

## Figures and Tables

**Figure 1 f1:**
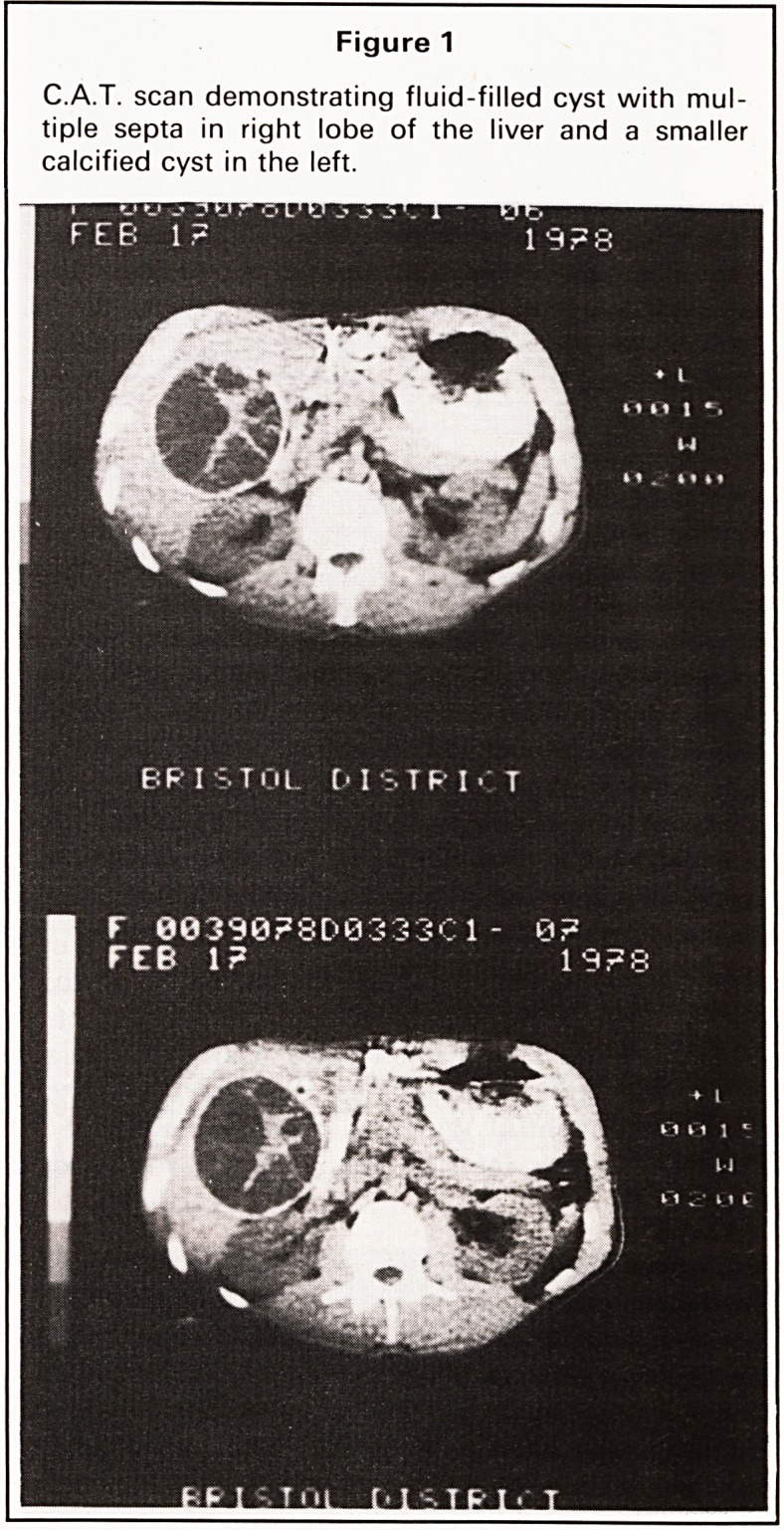


**Figure 2 f2:**
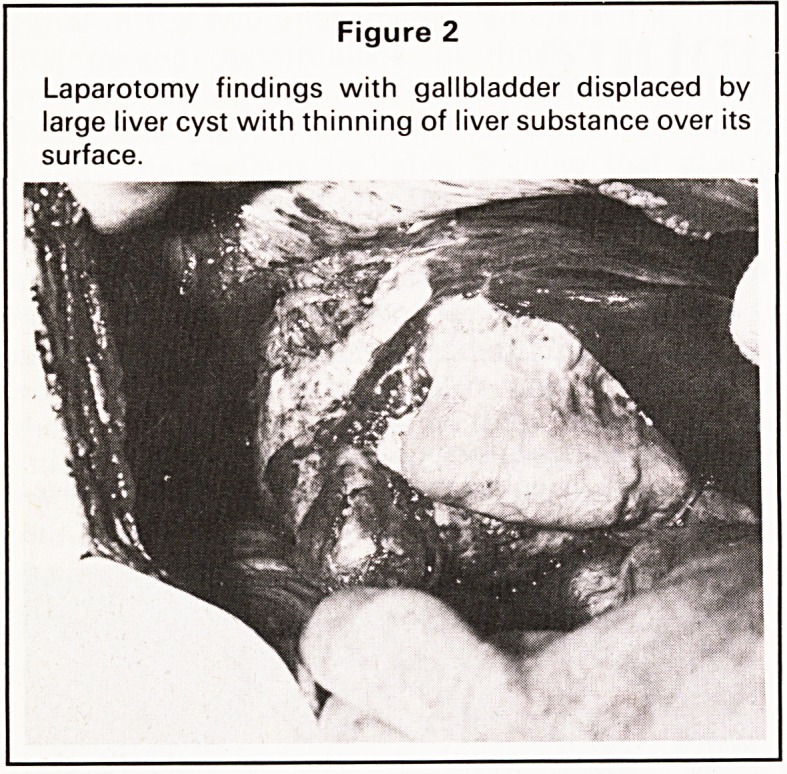


**Figure 3 f3:**
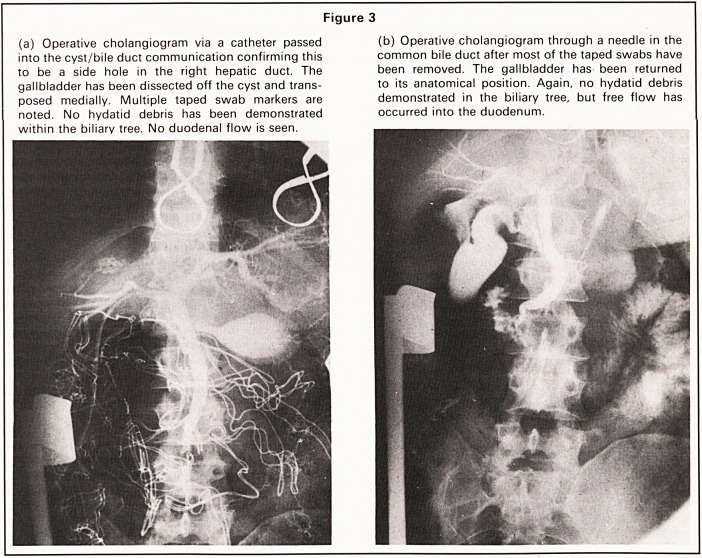


**Figure 4 f4:**
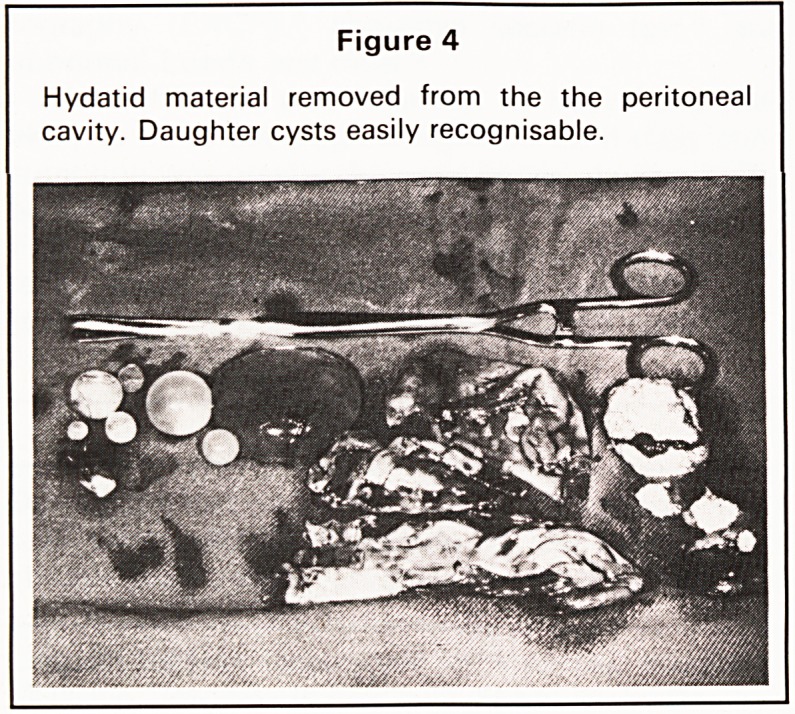


**Figure 5 f5:**